# Regulation of ventilatory sensitivity and carotid body proliferation in hypoxia by the PHD2/HIF‐2 pathway

**DOI:** 10.1113/JP271050

**Published:** 2015-10-06

**Authors:** Emma J. Hodson, Lynn G. Nicholls, Philip J. Turner, Ronan Llyr, James W. Fielding, Gillian Douglas, Indrika Ratnayaka, Peter A. Robbins, Christopher W. Pugh, Keith J. Buckler, Peter J. Ratcliffe, Tammie Bishop

**Affiliations:** ^1^ Wellcome Trust Centre for Human Genetics, Roosevelt DriveUniversity of OxfordOxfordOX3 7BNUK; ^2^ Ludwig Institute for Cancer Research, Roosevelt DriveUniversity of OxfordOxfordOX3 7DQUK; ^3^ Department of Physiology, Anatomy and Genetics, Sherrington Building, South Parks RoadUniversity of OxfordOxfordOX1 3PTUK

## Abstract

**Key points:**

- Sustained hypoxic exposure increases ventilatory sensitivity to hypoxia as part of physiological acclimatisation.
- Oxygen‐sensitive signals are transduced in animal cells by post‐translational hydroxylation of transcription factors termed hypoxia‐inducible factors (HIFs).
- Mice heterozygous for the principal ‘oxygen‐sensing’ HIF hydroxylase PHD2 (prolyl hydroxylase domain 2) show enhanced ventilatory sensitivity to hypoxia.
- To analyse the underlying mechanisms, functional (hypoxic ventilatory responses, HVRs) and anatomical (cellular proliferation within carotid bodies) responses were studied in genetic models of inducible and constitutive inactivation of PHD2 and its principal hydroxylation substrates, HIF‐1α and HIF‐2α.
- Inducible PHD2 inactivation enhanced HVR, similar to constitutive inactivation; both responses were almost entirely compensated for by specific inactivation of HIF‐2α.
- Inducible inactivation of HIF‐2α, but not HIF‐1α, strikingly reduced ventilatory acclimatisation to hypoxia and associated carotid body cell proliferation.
- These findings demonstrate a key role for PHD2 and HIF‐2α in ventilatory control and carotid body biology.

**Abstract:**

Ventilatory sensitivity to hypoxia increases in response to continued hypoxic exposure as part of acute acclimatisation. Although this process is incompletely understood, insights have been gained through studies of the hypoxia‐inducible factor (HIF) hydroxylase system. Genetic studies implicate these pathways widely in the integrated physiology of hypoxia, through effects on developmental or adaptive processes. In keeping with this, mice that are heterozygous for the principal HIF prolyl hydroxylase, PHD2, show enhanced ventilatory sensitivity to hypoxia and carotid body hyperplasia. Here we have sought to understand this process better through comparative analysis of inducible and constitutive inactivation of PHD2 and its principal targets HIF‐1α and HIF‐2α. We demonstrate that general inducible inactivation of PHD2 in tamoxifen‐treated *Phd2^f/f^;Rosa26^+/CreERT2^* mice, like constitutive, heterozygous PHD2 deficiency, enhances hypoxic ventilatory responses (HVRs: 7.2 ± 0.6 *vs*. 4.4 ± 0.4 ml min^−1^ g^−1^ in controls, *P* < 0.01). The ventilatory phenotypes associated with both inducible and constitutive inactivation of PHD2 were strongly compensated for by concomitant inactivation of HIF‐2α, but not HIF‐1α. Furthermore, inducible inactivation of HIF‐2α strikingly impaired ventilatory acclimatisation to chronic hypoxia (HVRs: 4.1 ± 0.5 *vs*. 8.6 ± 0.5 ml min^−1^ g^−1^ in controls, *P* < 0.0001), as well as carotid body cell proliferation (400 ± 81 *vs*. 2630 ± 390 bromodeoxyuridine‐positive cells mm^−2^ in controls, *P* < 0.0001). The findings demonstrate the importance of the PHD2/HIF‐2α enzyme–substrate couple in modulating ventilatory sensitivity to hypoxia.

## Key points

- Sustained hypoxic exposure increases ventilatory sensitivity to hypoxia as part of physiological acclimatisation.
- Oxygen‐sensitive signals are transduced in animal cells by post‐translational hydroxylation of transcription factors termed hypoxia‐inducible factors (HIFs).
- Mice heterozygous for the principal ‘oxygen‐sensing’ HIF hydroxylase PHD2 (prolyl hydroxylase domain 2) show enhanced ventilatory sensitivity to hypoxia.
- To analyse the underlying mechanisms, functional (hypoxic ventilatory responses, HVRs) and anatomical (cellular proliferation within carotid bodies) responses were studied in genetic models of inducible and constitutive inactivation of PHD2 and its principal hydroxylation substrates, HIF‐1α and HIF‐2α.
- Inducible PHD2 inactivation enhanced HVR, similar to constitutive inactivation; both responses were almost entirely compensated for by specific inactivation of HIF‐2α.
- Inducible inactivation of HIF‐2α, but not HIF‐1α, strikingly reduced ventilatory acclimatisation to hypoxia and associated carotid body cell proliferation.
- These findings demonstrate a key role for PHD2 and HIF‐2α in ventilatory control and carotid body biology.

**Abbreviations**
BrdU: bromodeoxyuridine
CB: carotid body
CH: chronic hypoxia
HIF: hypoxia‐inducible factor
HVR: hypoxic ventilatory response
PHD: prolyl hydroxylase domain
TH: tyrosine hydroxylase
VAH: ventilatory acclimatisation to hypoxia
VHL: von Hippel–Lindau protein

## Introduction

The control of breathing is a fundamental component of oxygen homeostasis, and ventilation increases within seconds in response to hypoxaemia. This acute hypoxic ventilatory response (HVR) defends against hypoxia, but is limited by hyperventilation‐induced hypocapnia. With exposure to more prolonged hypoxia, a further progressive increase in ventilation and arterial oxygenation develops over a period of hours to days in spite of the hypocapnia. This secondary response, often referred to as ventilatory acclimatisation to hypoxia (VAH; reviewed by Robbins, 2007), is associated with an increase in chemoreceptor sensitivity and, whilst usually associated with adaptation to altitude, is also important in diseases associated with chronic hypoxaemia. Despite intense study in many species, the mechanisms underlying chemoreceptor acclimatisation remain largely unknown. An understanding of this process could, however, represent an important target for therapeutic control of chemoreceptor activity.

Molecular insights into the regulation of gene expression by the hypoxia‐inducible factor (HIF) system have generated new opportunities for the understanding of such physiological responses to hypoxia (reviewed by Kaelin & Ratcliffe, 2008; Prabhakar & Semenza, 2012; Ratcliffe, 2013). HIF is an α/β heterodimeric transcription factor whose α subunits are regulated by oxygen levels through post‐translational hydroxylation of specific amino acid residues. The most important of these is the prolyl hydroxylation of residues that promote association of HIF‐α proteins with von Hippel–Lindau protein (pVHL) ubiquitin ligase and their subsequent proteasomal degradation. HIF prolyl hydroxylation is catalysed by the prolyl hydroxylase domain (PHD) enzymes, a series of closely related enzymes belonging to the 2‐oxoglutarate‐dependent dioxygenase family. A fall in oxygen availability impairs prolyl hydroxylation allowing HIF‐α proteins to escape destruction and form the transcriptional complex.

The HIF hydroxylase system is conserved throughout the animal kingdom, consisting of a single PHD and HIF‐α in the simplest animal *Trichloplax*, but undergoing whole genome duplications and deletions during vertebrate evolution, resulting in multiple copies of both HIF‐α (of which 1 and 2 are the best characterised) and PHD (1, 2 and 3) in mammals (Loenarz *et al*. 2010). Although PHD2 is the most abundant enzyme and the main regulator of HIF‐1α (Berra *et al*. 2003; Appelhoff *et al*. 2004), all PHD enzymes are able to regulate both HIF‐1α and HIF‐2α, with PHD1 and 3 having relatively greater action on HIF‐2α in some cell lines. Transcriptional profiling of HIF‐1α and HIF‐2α at the cellular level indicates that they activate distinct, although partially overlapping, sets of target genes (Mole *et al*. 2009; Schödel *et al*. 2012), generating interest in understanding the existence and nature of their differentiated physiological functions (Keith *et al*. 2012; Prabhakar & Semenza, 2012). Interestingly, despite HIF‐1α being the principal target of PHD2 in cell lines, studies in intact animals have identified a specific role for the PHD2/HIF‐2α couple in the regulation of erythropoiesis (Arsenault *et al*. 2013; Franke *et al*. 2013), a major component of acclimatisation to altitude.

The PHD/HIF system has also been shown to play an important part in ventilatory acclimatisation (Kline *et al*. 2002; Peng *et al*. 2006, 2011; Bishop *et al*. 2013; Yuan *et al*. 2013). Studies to date have investigated ventilatory control in mice with heterozygous loss of *Phd2*, *Hif‐1α* and *Hif‐2α*, which are viable and have no major phenotype in normoxia (Yu *et al*. 1999; Brusselmans *et al*. 2003; Aragones *et al*. 2008; Mazzone *et al*. 2009) – unlike their homozygous counterparts which are embryonic lethal (Carmeliet *et al*. 1998; Iyer *et al*. 1998; Kotch *et al*. 1999; Peng *et al*. 2000; Compernolle *et al*. 2002, 2003; Takeda *et al*. 2006) or, in the case of *Hif‐2α^−/−^* mice, develop gross abnormalities even if they survive embryonic development (Compernolle *et al*. 2002; Scortegagna *et al*. 2003). For instance, *Phd2^+/–^* mice show enhanced HVR similar to that observed after chronic exposure to hypoxia and overgrowth of the carotid body (CB) (Bishop *et al*. 2013). However, the PHD/HIF system also has profound effects on development, including that of sympathoadrenal and neuroendocrine systems that are relevant to ventilatory control (Tian *et al*. 1998; Compernolle *et al*. 2003; Bishop *et al*. 2008; Pan *et al*. 2012; Kenchegowda *et al*. 2014). The findings in constitutively heterozygous *Phd2^+/−^* mice therefore raise important questions as to the extent to which these effects are developmental as opposed to a reflection of adaptive effects of hypoxia on the activity of PHD2, and which targets (HIF‐α proteins or other proposed PHD2 substrates, e.g. Takahashi *et al*. 2011) mediate the observed effects.

To address these questions we have performed comparative studies of multiple genetic models that reflect both inducible (acute) inactivation of PHD2 and its principal HIF‐α targets: HIF‐1α and HIF‐2α, and constitutive (chronic) heterozygous inactivation of PHD2 and HIF‐1/2α. Our findings reveal that acute inducible inactivation of PHD2 increases ventilatory sensitivity to hypoxia and that these effects are strongly dependent on the integrity of HIF‐2α. HIF‐2α was also critical for both the enhanced HVR, and the cellular proliferation within the CB, which were observed in response to a 7‐day exposure to chronic hypoxia. These effects were reflected in the association of HIF‐2α heterozygosity with reduced ventilatory sensitivity, both in the context of PHD2 heterozygosity and after chronic hypoxia. Together our findings implicate the PHD2/HIF‐2 couple as the dominant mediator of enhanced HVR during ventilatory acclimatisation to hypoxia, at least under the conditions of our experiments.

## Methods

### Animals

All animal procedures were compliant with the UK Home Office Animals (Scientific Procedures) Act 1986 and Local Ethical Review Procedures (University of Oxford Medical Sciences Division Ethical Review Committee). The authors understand the ethical principles of *The Journal of Physiology* and all work was conducted in compliance with stated standards (Grundy, 2015). Male mice, approximately 3 months old and from the same litter, were used for all comparisons, unless stated otherwise. *Phd2^f/f^*, *Hif‐1α^f/f^* and *Hif‐2α^f/f^* (where f denotes the floxed allele) conditional knockout and *Rosa26Cre^ERT2^* mice have all been described previously and were obtained from these sources (Vooijs *et al*. 2001; Cramer *et al*. 2003; Higgins *et al*. 2004; Gruber *et al*. 2007; Mazzone *et al*. 2009). Each line had been backcrossed with C57BL/6 for at least five generations (Adam *et al*. 2011) and was intercrossed to generate littermates of appropriate genotypes. *Phd2^+/−^*, *Hif‐1α^+/−^* and *Hif‐2α^+/–^* mice are as described previously (Carmeliet *et al*. 1998; Brusselmans *et al*. 2003; Mazzone *et al*. 2009); these lines were intercrossed to generate mice that were maintained on a mixed Swiss/129/SvEv/C57BL/6 genetic background. Genotype was determined by PCR (details on request). Mice were housed in individually ventilated cages with free access to food and water.

### Tamoxifen administration

Tamoxifen (prepared to 20 mg ml^−1^ in corn oil; Sigma, St Louis, MO, USA) was administered by oral gavage to ∼6‐week‐old mice at a dose of 2 mg day^–1^ for five consecutive days as described by Arsenault *et al*. (2013). Recombination was assessed using DNA isolated from ear biopsies obtained 10 days after the first dose of tamoxifen (PCR primers used to test for recombination are as described by Adam *et al*. 2011). Ventilation measurements were also taken (and chronic hypoxia treatment started) at this timepoint, i.e. 10 days after the first tamoxifen dose, to allow for assessment of ventilatory responses prior to the onset of other steady‐state, long‐term effects of PHD2/HIF inactivation such as polycythaemia.

### Plethysmography

Tidal volume and respiratory rate were measured in awake, unrestrained mice using individual whole body plethysmographs (600 ml volume; Model PLY4211, Buxco, DSI, St. Paul, MI, USA). Minute ventilation was calculated from tidal volume and respiratory rate. Ventilatory parameters were derived using FinePointe software (Buxco) and adjusted to body weight as measured immediately prior to plethysmography. Premixed gas was delivered to each chamber as described previously (Bishop *et al*. 2013). The acute hypoxic stimuli consisted of 10% oxygen, balance nitrogen or 10% oxygen, 3% carbon dioxide, balance nitrogen. The HVR to each stimulus was defined as the difference between minute ventilation (or tidal volume or respiratory rate; see Table 1) during the 1 min prior to the onset of hypoxia and the first 1 min of stable hypoxia (i.e. excluding the first 30 s of hypoxia).

**Table 1:** **Acute hypoxic ventilatory responses after conditional *Phd*2 inactivation in adult mice**

		Hypoxic ventilatory response		
	Acute hypoxic stimulus	*Phd2^f/f^*	*Phd2^f/f^;CreER*	*P* value
Minute ventilation (ml min^−1^ g^−1^)	10% O_2_/3% CO_2_	4.44 ± 0.44	7.19 ± 0.60	**0.003**
	10% O_2_	1.52 ± 0.40	2.18 ± 0.46	0.286
Tidal volume (ml g^−1^)	10% O_2_/3% CO_2_	9.11 ± 0.53	15.14 ± 1.10	**<0.001**
	10% O_2_	2.83 ± 0.40	4.84 ± 0.51	**0.009**
Respiratory rate (breaths min^−1^)	10% O_2_/3% CO_2_	98.9 ± 42.8	97.53 ± 18.6	0.975
	10% O_2_	47.0 ± 34.7	49.1 ± 27.3	0.961

Minute ventilation, tidal volume and respiratory rate in response to the indicated acute hypoxic stimulus, following tamoxifen‐induced recombination. Mean ± SEM; *n* = 6 littermate pairs; *P* < 0.05 highlighted in bold type.

### Chronic hypoxic exposure of mice and bromodeoxyuridine labelling

Mice were housed in a normobaric chamber containing 10% oxygen for 7 days with controlled temperature, humidity and carbon dioxide levels, and free access to food and water, as described previously (Bishop *et al*. 2013) and removed from this chamber only for plethysmography testing at 48 h. All mice were 20–35 g body weight at the start of the procedure. Mice were given 50 mg kg^−1^ bromodeoxyuridine (BrdU, Sigma) by intraperitoneal injection immediately prior to the start of hypoxia exposure (day 0) followed by supplementation of drinking water with 1 mg ml^−1^ BrdU until termination of the experiment, as described previously (Pardal *et al*. 2007; Bishop *et al*. 2013).

### Blood measurements

Blood was taken from the tail vein of mice using heparinised capillary tubes, and haematocrits were measured using a haematocrit centrifuge (model C‐MH30, Unico, Dayton, NJ, USA).

### Immunohistochemistry

Mice were killed by overdose of isoflurane and exsanguination followed by immediate dissection of CBs and fixation in 4% paraformaldehyde/PBS overnight prior to transfer into 70% ethanol and processing. Paraffin‐embedded tissue samples were sectioned to 4 μm thickness and immunostained with a polyclonal anti‐tyrosine hydroxylase (TH) antibody (Novus Biologicals, Abingdon, UK) as previously described (Bishop *et al*. 2013), or with an anti‐BrdU antibody according to the manufacturer's instructions (Becton Dickinson, Oxford, UK). To quantify BrdU‐positive cells, the density of positively stained cells was estimated using Image J software; adjacent sections were immunostained for TH to delineate the CB. Stereological analyses of TH‐positive cell density, TH‐positive cell number and CB volume were performed using Image J software on every fourth section throughout CBs harvested from mice 8–9 weeks after tamoxifen treatment.

### Statistical analysis

Data are shown as mean ± SEM. Statistical analyses were performed using unpaired Student's *t* tests. For repeated measures, data were analysed by ANOVA, followed by Tukey's test or *t *tests with Holm–Sidak correction for multiple comparisons, as appropriate. *P* values < 0.05 were considered significant.

## Results

### Enhanced ventilatory responses after conditional PHD2 inactivation in adult mice

**Figure 1:**
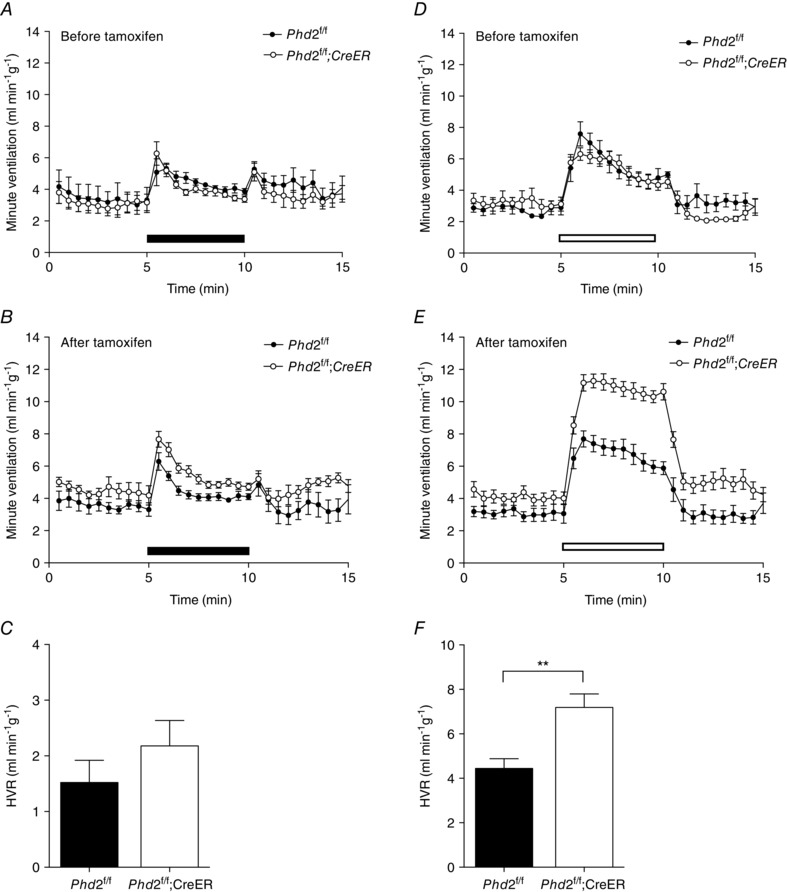
**Acute hypoxic ventilatory responses before and after conditional *Phd*2 inactivation in adult mice** Minute ventilation before, during and after an acute hypoxic exposure to 10% oxygen (*A–C*, closed bars), or 10% oxygen with added 3% carbon dioxide (*D–F*, open bars) in littermate controlled *Phd2* ^f/f^ *versus Phd2^f/f^*;*CreER* mice. Animals studied before (A and D) and 10 days after (B and E) starting tamoxifen treatment. Hypoxic ventilatory responses (HVRs) calculated from *B* and *E* are shown in *C* and *F*, respectively. Mean ± SEM, *n* = 6 littermate pairs; **P < 0.01.

To better define the role of PHD2 in regulating ventilatory acclimatisation we first examined the effect of inducible inactivation of PHD2 on the HVR using *Phd2^f/f^;Rosa26^+/CreERT2^* (here after referred to as *Phd2^f/f^;CreER*) mice. Administration of tamoxifen at ∼6 weeks of age causes general inactivation of PHD2 by Cre recombinase‐mediated excision of sequences encoding the PHD2 catalytic domain. Ventilation was analysed in conscious, unrestrained *Phd2^f/f^;CreER* mice by whole animal plethysmography before, during and after a 5 min acute hypoxic stimulus both before and after (measured 10 days after the first dose) tamoxifen treatment. Responses were compared with those of littermate control *Phd2^f/f^* mice lacking the *Rosa26^CreERT2^* allele (Fig. 1; Table 1).

In control *Phd2^f/f^* animals prior to tamoxifen treatment, acute exposure to hypoxia (10% oxygen) elicited an immediate but poorly sustained HVR (Fig. 1 *A*). The response to unregulated breathing of a hypoxic atmosphere decreases as hyperventilation induces respiratory alkalosis, which in turn depresses ventilation. This can be offset by adding a small concentration of carbon dioxide to the hypoxic atmosphere used for acute stimulation. Thus, control mice exposed to 10% oxygen with 3% carbon dioxide (as described by Bishop *et al*. 2013; Slingo *et al*. 2014), and based on published data which show that adding 3% carbon dioxide prevents a major rise in arterial pH or fall in P aC O2 when mice are exposed to 7% oxygen (Ishiguro *et al*. 2006), exhibited a more sustained HVR (Fig. 1 *D*) than when exposed to 10% oxygen alone (Fig. 1 *A*). Treatment of the control *Phd2^f/f^* mice with tamoxifen did not alter HVR with either 10% oxygen or 10% oxygen with 3% carbon dioxide (Fig. 1 *B vs*. *A*, *E vs*. *D*).

In *Phd2^f/f^;CreER* mice, acute exposure to hypoxia (either 10% oxygen or 10% oxygen with 3% carbon dioxide) elicited HVRs of similar magnitude to those of littermate control *Phd*2^f/f^ mice when tested prior to tamoxifen treatment (Fig. 1 *A*, *D*). Ten days after the first dose of tamoxifen genomic recombination was efficient (data not shown) and mice appeared healthy, without visible abnormalities. Ventilatory responses to 10% oxygen and 10% oxygen with 3% carbon dioxide were again tested. Large increases in HVR were observed in *Phd2^f/f^;CreER* mice compared to their *Phd2^f/f^* littermate controls. Responses were particularly clearly observed in the sustained HVR observed in response to breathing 10% oxygen with 3% carbon dioxide (Fig. 1 *E*; Table 1). These were similar in magnitude and character to those observed after 7 days of pre‐exposure to hypoxia (Bishop *et al*. 2013, and see below) and primarily involved an increase in tidal volume (Table 1). Furthermore, this was accompanied by a small but significant increase in basal minute ventilation (in 21% oxygen) in *Phd2^f/f^;CreER* mice compared to *Phd2^f/f^* controls (4.09 ± 0.3 *vs*. 3.10 ± 0.3 ml min^−1^ g^−1^, *P* = 0.03). These findings reveal that induced, general loss of PHD2 enhances the acute ventilatory response to hypoxia, and over a comparable period of time to ventilatory acclimatisation to hypoxia, consistent with the hypothesis that hypoxic inhibition of PHD2 plays a key mechanistic role in this process.

### Combined inactivation of PHD2 and HIF‐α isoforms

As PHD2 regulates both HIF‐1α and HIF‐2α and has been reported to hydroxylate many non‐HIF substrates, we next analysed whether the PHD2‐dependent ventilatory phenotypes are mediated through HIF regulation and whether they are specifically dependent on a particular HIF‐α isoform.

**Figure 2:**
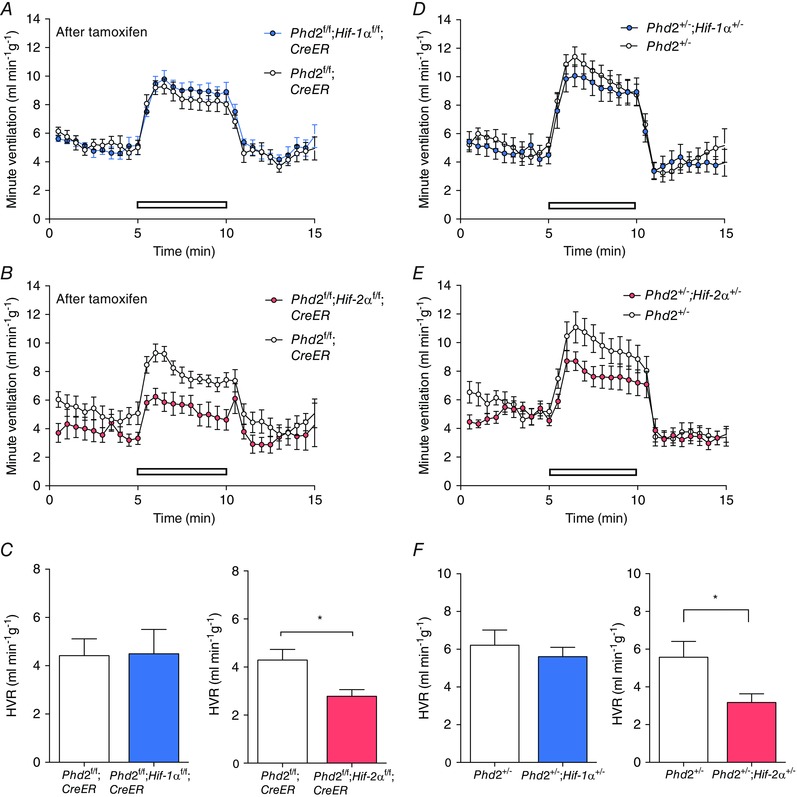
**Effect of conditional or constitutive inactivation of *Hif‐1α* or *Hif‐2α* on hypoxic ventilatory responses of mice with associated inactivation of *Phd*2** Minute ventilation before, during and after an acute hypoxic exposure to 10% oxygen with 3% carbon dioxide (open bars) in: *A*, tamoxifen‐treated *Phd2^f/f^;CreER versus Phd2^f/f^*;*Hif‐1α^f/f^;CreER* (*n* = 6 pairs); *B*, tamoxifen‐treated *Phd2^f/f^;CreER versus Phd2^f/f^;Hif‐2α^f/^* ^f^;CreER (*n* = 5 pairs); *D*, *Phd2^+/−^ versus Phd2^+/−^*;*Hif‐1α^+/−^* mice (*n* = 7 pairs); and *E*, *Phd2^+/−^ versus Phd2^+/−^*;*Hif‐2α^+/−^* mice (*n* = 7 pairs). Calculated HVRs for data shown in *A–B* and *D–E* are shown in *C* and *F*, respectively. Mean ± SEM; *P < 0.05.

In the first instance, we tested this by combining acute general inactivation of PHD2 with that of either HIF‐1α or HIF‐2α by breeding *Phd2^f/f^;Hif‐1α^f/f^;CreER* or *Phd2^f/f^;Hif‐2α^f/f^;CreER* mice and comparing ventilatory responses to those of *Phd2^f/f^;CreER* control mice, after treatment with tamoxifen. HVR was measured in response to both 10% oxygen and 10% oxygen with 3% carbon dioxide. Prior to tamoxifen treatment, similar HVR values were obtained in all mice, regardless of genotype (data not shown). As previously, ventilatory responses were re‐measured 10 days after the first dose of tamoxifen, at which point efficient genomic recombination had taken place (data not shown), but other systemic responses such as changes in haematocrit had not developed. Effects were again more clearly observed on the sustained HVR observed with 10% oxygen and 3% carbon dioxide. HVRs to this stimulus were significantly reduced in *Phd2^f/f^;Hif‐2α^f/f^;CreER* mice but not *Phd2^f/f^;Hif‐1α^f/f^;CreER* mice when compared to *Phd2^f/f^;CreER* control mice (Fig. 2 *A–C*; Table 2). There were no significant effects on basal ventilation in either case, despite a trend towards reduced ventilation in *Phd2^f/f^;Hif‐2α^f/f^;CreER* mice. The results indicate that the acute ventilatory effects of PHD2 inactivation can be largely reversed through concomitant acute inactivation of HIF‐2α but not HIF‐1α.

**Table 2:** **Ventilatory responses after conditional inactivation of *Phd2* and concomitant inactivation of *Hif‐1α* or *Hif‐2α***

	Hypoxic ventilatory response (ml min^−1^ g^−1^)		
Acute hypoxic stimulus	*Phd2^f/f^;CreER*	*Phd2^f/f^*;*Hif‐1α^f/f^;CreER*	*P* value
10% O_2_/3% CO_2_	4.42 ± 0.69	4.49 ± 1.01	0.95
10% O_2_	0.59 ± 0.77	0.45 ± 0.27	0.86
	***Phd2^f/f^;CreER***	***Phd2^f/f^*;*Hif‐2α^f/f^;CreER***	
10% O_2_/3% CO_2_	4.29 ± 0.44	2.78 ± 0.28	**0.02**
10% O_2_	1.58 ± 0.86	–0.98 ± 0.57	0.15

Acute hypoxic ventilatory responses of tamoxifen‐treated *Phd2^f/f^;CreER versus Phd2^f/f^*;*Hif‐1α^f/f^;CreER* mice (*n* = 6 pairs) or tamoxifen‐treated *Phd2^f/f^;CreER versus Phd2^f/f^*;*Hif‐2α^f/f^;CreER* mice (*n* = 5 pairs). Values shown are mean changes in minute ventilation (± SEM) in response to the indicated acute hypoxic stimulus. *P* < 0.05 highlighted in bold type.

After longer periods of PHD2 inactivation these interventions have been reported to result in major changes in haematopoiesis. To enable comparison of our work with those reports, we measured haematocrits 6 weeks following tamoxifen treatment. In line with previous reports (Takeda *et al*. 2007, 2008; Minamishima *et al*. 2008), *Phd2^f/f^;CreER* mice developed severe polycythaemia (Table 6A) and hepatosplenomegaly (data not shown). Concomitant inactivation of HIF‐2α (tamoxifen‐treated *Phd2^f/f^;Hif‐2α^f/f^;CreER* mice), but not HIF‐1α (tamoxifen‐treated *Phd2^f/f^;Hif‐1α^f/f^;CreER* mice), ablated the development of polycythaemia (Table 6A). These findings are thus in line with previous findings using similar but not identical mouse lines (Arsenault *et al*. 2013; Franke *et al*. 2013), and confirm the dominant role of HIF‐2α in regulating erythropoiesis.

Ventilation measurements in these mice were made prior to the onset of the polycythaemia when mice appeared healthy, at the earliest time point following tamoxifen treatment when recombination could be confirmed. Nevertheless, these models may be confounded by, for example, changes in iron status that precede the onset of polycythaemia. To confirm the findings on the HIF‐α isoform dependence of PHD2‐dependent increases in HVR in an independent genetic model, we analysed the HIF‐α dependence of the ventilatory effects observed in *Phd2^+/−^* mice (Bishop *et al*. 2013) using double heterozygotes (*Phd2^+/−^;Hif‐1α^+/−^* or *Phd2^+/−^;Hif‐2α^+/–^* animals). These mice developed normally and were essentially indistinguishable from wild‐type mice under normoxic conditions; haematocrits were normal in *Phd2^+/−^* mice, with *Phd2^+/−^;Hif‐1α^+/−^* and *Phd2^+/−^;Hif‐2α^+/−^* mice being only very mildly polycythaemic/anaemic, respectively, when compared to *Phd2^+/−^* mice (Table 6A).

The required intercrosses generated mice on a mixed genetic background rather than the C57BL/6 background on which we had previously observed enhanced HVR in *Phd2^+/−^* animals (Bishop *et al*. 2013), and HVR has been reported to vary with genetic background (Han *et al*. 2002; Balbir *et al*. 2007). For those reasons, we first confirmed enhanced HVRs in *Phd2^+/−^* versus wild‐type littermate mice (data not shown). The effect of heterozygous inactivation of HIF‐1α or HIF‐2α on the *Phd*2*^+/−^* ventilatory responses was tested by directly comparing HVRs in *Phd*2*^+/–^* mice with littermates that were also heterozygous for the different HIF‐α isoforms. HVRs were significantly reduced in *Phd2^+/−^;Hif‐2α^+/−^ versus Phd2^+/−^* mice, but not in the equivalent HIF‐1α comparison (Fig. 2 *D–F*; Table 3). The findings are therefore consistent with those following tamoxifen‐inducible general inactivation. Furthermore, the results demonstrate that although ventilatory control and erythropoiesis are both strongly regulated by PHD2 and HIF‐2α, the two processes can be uncoupled from each other, with changes in HVR occurring independently of erythropoietic stimulation, in the different settings.

**Table 3:** **Acute hypoxic ventilatory responses of *Phd2^+/^^−^ versus Phd*2*^+/^^−^*;*Hif‐1α^+/^^−^* mice or *Phd2^+/^^−^* versus *Phd2^+/^^−^;Hif‐2α^+/^^−^* mice**

	Hypoxic ventilatory response (ml min^−1^ g^−1^)		
Acute hypoxic stimulus	*Phd2^+/−^*	*Phd*2*^+/−^*;*Hif‐1α^+/−^*	*P* value
10% O_2_/3% CO_2_	6.21 ± 0.81	5.61 ± 0.49	0.54
10% O_2_	2.04 ± 0.64	1.29 ± 0.38	0.33
	***Phd2^+/−^***	***Phd2^+/−^;Hif‐2α^+/−^***	
10% O_2_/3% CO_2_	5.58 ± 0.83	3.17 ± 0.46	**0.03**
10% O_2_	2.97 ± 0.73	1.26 ± 0.45	0.07

Values shown are mean changes in minute ventilation (± SEM) in response to the indicated acute hypoxic stimulus; *n* = 7 littermate pairs for each genotype; *P* < 0.05 highlighted in bold type.

### A major role for HIF‐2α in ventilatory acclimatisation to hypoxia

**Figure 3:**
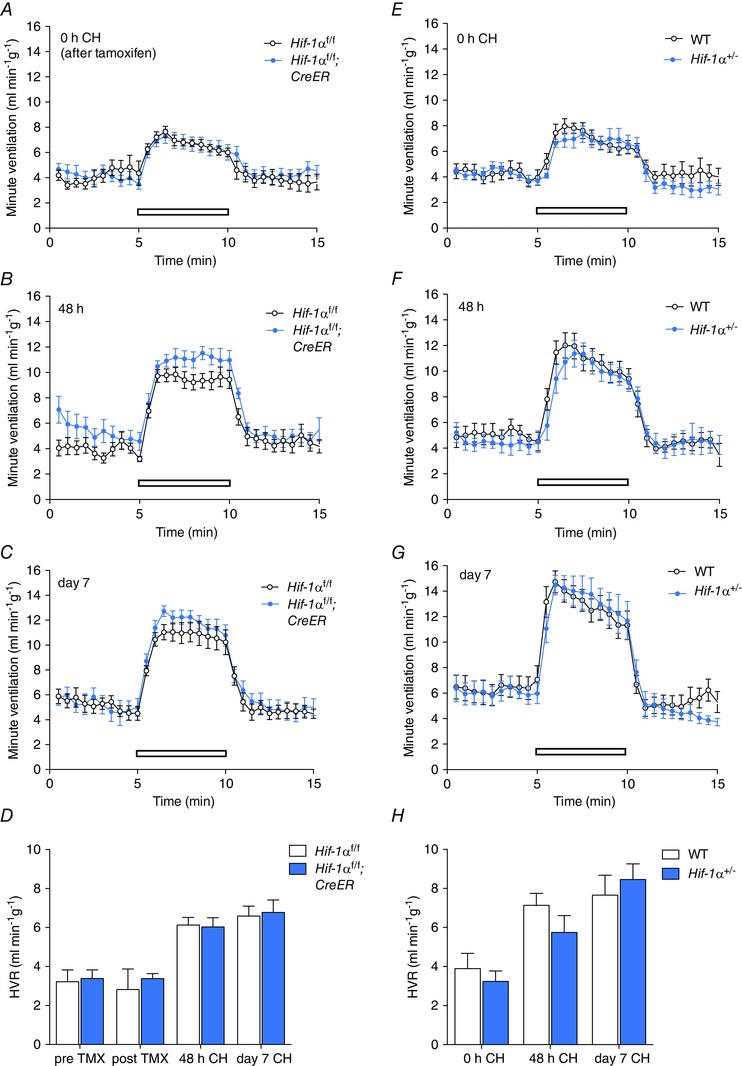
**Effect of conditional or constitutive inactivation of *Hif‐1α* on the hypoxic ventilatory response in mice before and after ventilatory acclimatisation to chronic hypoxia** Minute ventilation before, during and after an acute hypoxic exposure to 10% oxygen with 3% carbon dioxide (open bars). Comparisons were tamoxifen‐treated *Hif‐1α^f/f^;CreER versus Hif‐1α^f/f^* littermates (*n* = 7 pairs)(A–D); *Hif‐1α^+/−^ versus* wild‐type littermates (*n* = 6 pairs) (E–H). Animals were studied immediately before (0 h, *A*, *E*) or after (48 h, *B*, *F*; 7 days, *C*, *G*) exposure to chronic hypoxia (CH). *D* and *H*, hypoxic ventilatory responses (HVR) calculated from changes in minute ventilation before and after exposure to CH for the indicated times. For mice bearing the conditionally inactivated allele, HVR values are shown before and after tamoxifen (TMX). Values shown are mean ± SEM.

**Figure 4:**
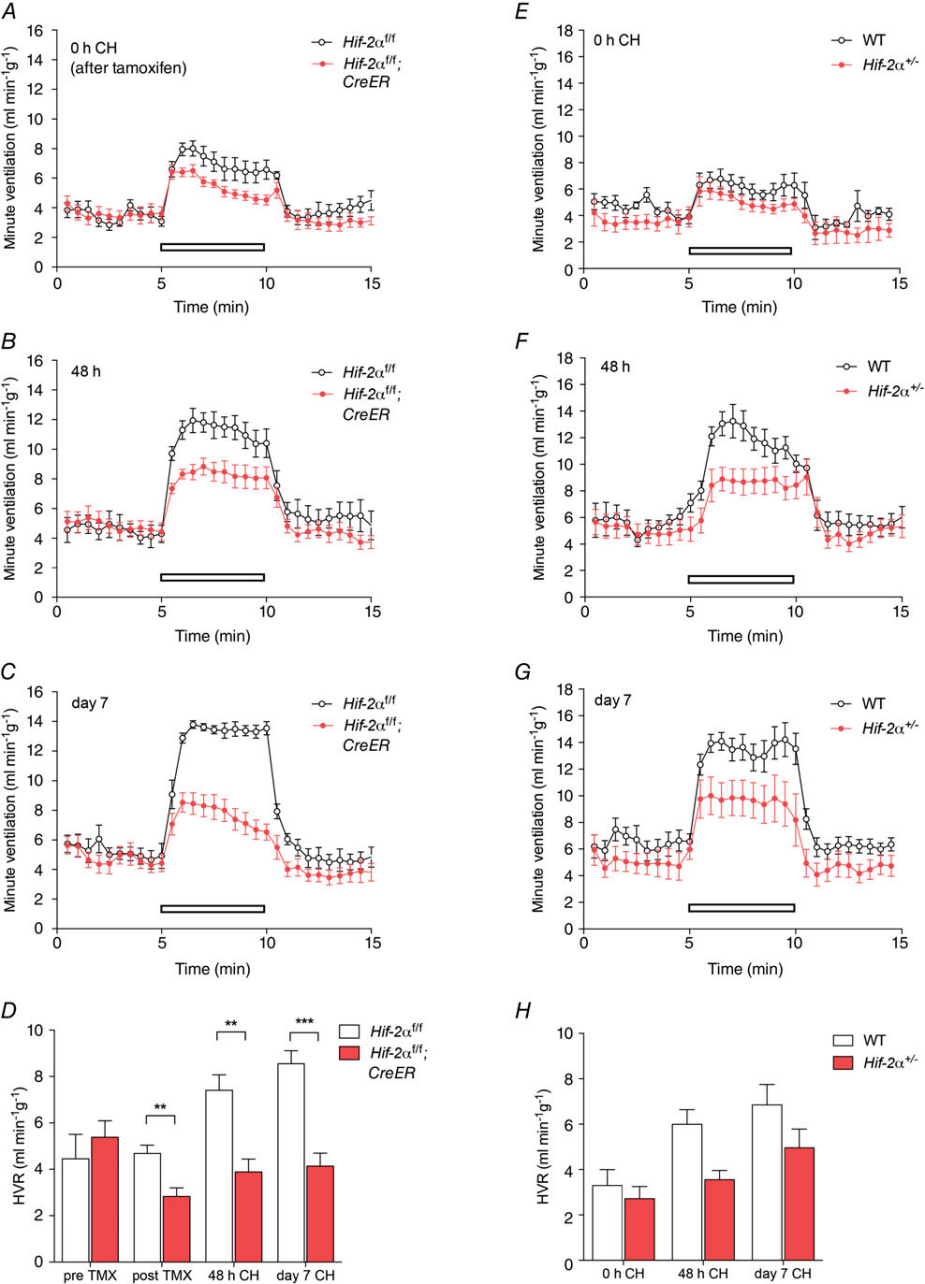
**Effect of conditional or constitutive inactivation of *Hif‐2α* on the hypoxic ventilatory response in mice before and after ventilatory acclimatisation to chronic hypoxia** Minute ventilation before, during and after an acute hypoxic exposure to 10% oxygen with 3% carbon dioxide (open bars). Comparisons were tamoxifen‐treated *Hif‐2α^f/f^;CreER versus Hif‐2α^f/f^* littermates (*n* = 7 pairs) (*A–D*); *Hif‐2α^+/−^ versus* wild‐type littermates (*n* = 6 pairs) (*E–H*). Animals were studied immediately before (0 h, *A*, *E*) or after (48 h, *B*, *F*; 7 days, *C*, *G*) exposure to chronic hypoxia (CH). *D* and *H*, hypoxic ventilatory responses (HVR) calculated from changes in minute ventilation before and after exposure to CH for the indicated times. For mice bearing the conditionally inactivated allele, HVR values are shown before and after tamoxifen (TMX). Mean ± SEM; **P < 0.01, ***P < 0.001.

Given that our findings implicate HIF‐2α in mediating increased HVR in the different models of PHD2 inactivation, we proceeded to test the role of the different HIF‐α isoforms in enhanced HVR following pre‐exposure to hypoxia. This was done by measuring HVR in mice after inactivation of HIF‐1α or HIF‐2α using both the inducible and the constitutive systems, i.e. comparison of tamoxifen‐treated *Hif‐1α* or *Hif‐2α^f/f^;CreER versus Hif‐1α* or *Hif*‐*2α^f/f^* control mice and comparison of heterozygous *Hif‐1α* or *Hif‐2α* versus wild‐type mice (Figs 3 and 4). Animals were pre‐exposed to chronic hypoxia (10% oxygen in a normobaric chamber for 7 days), starting 10 days after the first tamoxifen dose in the case of the conditional system. HVR was measured before and after 48 h and 7 days at 10% oxygen.

Progressive respiratory acclimatisation was observed in control (i.e. wild‐type or tamoxifen‐treated *Hif‐1α^f/f^* and *Hif‐2α^f/f^*) mice, with a significant increase in HVR after 48 h at 10% oxygen, which was further enhanced after 7 days at 10% oxygen (Figs 3 and 4; Tables 4 and 5).

**Table 4:** **Acute hypoxic ventilatory responses of mice after conditional inactivation of *Hif‐1α* or *Hif‐2α* followed by chronic hypoxia exposure**

(*A*)					
		Hypoxic ventilatory response (ml min^−1^ g^−1^)			
Acute hypoxic stimulus	Chronic hypoxia duration	*Hif‐1α^f/f^*	*Hif‐1α^f/f^;CreER*	*t* test *P* value	ANOVA *P* value
10% O_2_/ 3% CO_2_	0 h	2.82 ± 1.05	3.38 ± 0.26	0.500	**<0.0001**
	48 h	6.13 ± 0.39	6.03 ± 0.48	0.901	
	7 days	6.59 ± 0.64	6.93 ± 0.47	0.682	
ANOVA *P* value		0.6305			0.8227
10% O_2_	0 h	0.99 ± 0.51	0.39 ± 0.22	0.358	**0.0015**
	48 h	2.58 ± 0.72	1.31 ± 0.38	0.053	
	7 days	2.27 ± 0.62	2.51 ± 0.36	0.290	
ANOVA *P* value		0.0699			0.6683
**(*B*)**					
		**Hypoxic ventilatory response (ml min^−1^ g^−1^)**			
**Acute hypoxic stimulus**	**Chronic hypoxia duration**	***Hif‐2α^f/f^***	***Hif‐2α^f/^*^f^*;CreER***	***t* test *P* value**	**ANOVA *P* value**
10% O_2_/ 3% CO_2_	0 h	4.68 ± 0.37	2.83 ± 0.36	**0.0060**	**<0.0001**
	48 h	7.41 ± 0.66	3.89 ± 0.55	**0.0022**	
	7 days	8.55 ± 0.48	4.14 ± 0.45	**0.0004**	
ANOVA *P* value		**0.0002**			**0.0212**
10% O_2_	0 h	0.73 ± 0.64	–0.13 ± 0.51	0.24479	**0.0013**
	48 h	2.10 ± 0.39	0.78 ± 0.76	0.08107	
	7 days	3.72 ± 0.27	1.16 ± 0.43	**0.00144**	
ANOVA *P* value		**0.0061**			0.2287

Hypoxic ventilatory responses of: *A*, *Hif‐1α^f/f^;CreER versus Hif‐1α^f/f^*; and *B*, *Hif‐2α^f/f^;CreER versus Hif‐2α^f/f^* mice after tamoxifen‐induced recombination (0 h), followed by 48 h and 7 days of exposure to 10% oxygen. Values shown are mean changes in minute ventilation (± SEM) in response to the indicated acute hypoxic stimulus; *n* = 7 littermate pairs for each genotype. Comparisons were made using two‐way ANOVAs (right‐hand column *P* value = chronic hypoxia factor; bottom row *P* value = genotype factor; right column, bottom row *P* value = chronic hypoxia/genotype interaction factor), followed by *t* tests (with Holm–Sidak correction) for analysis of individual time points; *P* < 0.05 highlighted in bold type.

**Table 5:** **Acute hypoxic ventilatory responses of *Hif‐1α^+/^^−^* or *Hif‐2α^+/^^−^* mice before and after chronic hypoxia exposure**

(*A*)					
		Hypoxic ventilatory response (ml min^−1^ g^−1^)			
Acute hypoxic stimulus	Chronic hypoxia duration	WT	*Hif‐1α^+/−^*	*t* test *P* value	ANOVA *P* value
10% O_2_/ 3% CO_2_	0 h	3.89 ± 0.79	3.24 ± 0.53	0.562	**0.0001**
	48 h	7.13 ± 0.62	5.74 ± 0.87	0.220	
	7 days	7.66 ± 1.02	8.46 ± 0.80	0.475	
ANOVA *P* value		0.4470			0.4363
10% O_2_	0 h	1.50 ± 0.31	1.20 ± 0.80	0.771	**0.0021**
	48 h	3.88 ± 0.75	2.63 ± 0.76	0.250	
	7 days	4.24 ± 0.98	4.86 ± 0.75	0.563	
ANOVA *P* value		0.5947			0.4986
**(*B*)**					
		**Hypoxic ventilatory response (ml min^−1^ g^−1^)**			
**Acute hypoxic stimulus**	**Chronic hypoxia duration**	**WT**	***Hif‐2α^+/−^***	***t* test *P* value**	**ANOVA *P* value**
10% O_2_/3% CO_2_	0 h	3.32 ± 0.70	2.41 ± 0.65	0.378	**<0.0001**
	48 h	5.99 ± 0.65	3.56 ± 0.40	0.020	
	7 days	6.85 ± 0.90	4.96 ± 0.82	0.067	
ANOVA *P* value		0.0527			0.3433
10% O_2_	0 h	2.18 ± 0.55	0.59 ± 0.88	0.129	**0.0009**
	48 h	3.61 ± 0.50	1.51 ± 0.63	**0.048**	
	7 days	5.43 ± 1.09	4.19 ± 0.45	0.233	
ANOVA *P* value		**0.0068**			0.8566

Hypoxic ventilatory responses of: *A*, *Hif‐1α^+/−^ versus* WT; and *B*, *Hif‐2α^+/−^ versus* WT mice after 0 h, 48 h and 7 days of exposure to 10% oxygen. Values shown are mean changes in minute ventilation (±SEM); *n* = 6 littermate pairs for each genotype. Comparisons were made using two‐way ANOVAs (right‐hand column *P* value = chronic hypoxia factor; bottom row *P* value = genotype factor; right column, bottom row *P* value = chronic hypoxia/genotype interaction factor), followed by *t* tests (with Holm–Sidak correction) for analysis of individual time points; *P* < 0.05 highlighted in bold type.

Neither acute inactivation of HIF‐1α in tamoxifen‐treated *Hif‐1α^f/f^;CreER* mice (compared to control, tamoxifen‐treated *Hif‐1α^f/f^*) nor constitutive loss of HIF‐1α in *Hif‐1α^+/−^* mice (compared to wild‐type littermates) significantly altered HVR measured over 48 h to 7 days at 10% oxygen (Fig. 3; Tables 4 *A* and 5 *A*), although there was a trend towards decreased HVRs in *Hif‐1α^f/f^;CreER* and *Hif‐1α^+/−^* mice after 48 h of hypoxia (Tables 4 *A* and 5 *A*). In contrast, acute inactivation of HIF‐2α (tamoxifen‐treated *Hif‐2α^f/f^;CreER* mice) and constitutive inactivation of HIF‐2α in *Hif‐2α^+/−^* mice significantly reduced the increases in HVR after 48 h or 7 days of hypoxia (Fig. 4; Tables 4 *B* and 5 *B*). Interestingly, effects on HVR at baseline (i.e. prior to chronic hypoxia pretreatment) were also observed in *Hif‐2α^f/f^;CreER* mice (Fig. 4A; Table 4 *B*) without any significant effect on basal ventilation in normoxia; a similar trend towards a reduction in HVRs was noted in normoxic *Hif‐2α^+/−^* mice but this did not reach statistical significance (Fig. 4 *E*; Table 5 *B*). Together these results demonstrate that, regardless of the genetic model, HIF‐2α plays a major role in ventilatory acclimatisation to hypoxia (and contributes to maintenance of basal HVR), consistent with dependence of the enhanced HVR on HIF‐2α in PHD2 deficient animals.

Although both *Hif‐2α^+/−^* and tamoxifen‐treated *Hif‐2α^f/f^;CreER* mice had reduced ventilatory acclimatisation to hypoxia, they differed in their erythropoietic status. Erythropoiesis was unaffected by loss of HIF‐2α or HIF‐1α in *Hif‐2α^+/−^* or *Hif‐1α^+/−^* mice (Table 6 *B*); likewise, it was unaffected in tamoxifen‐treated *Hif‐1α^f/f^;CreER* mice (Table 6 *B*). In contrast, hypoxia‐induced erythropoiesis was abrogated in tamoxifen‐treated *Hif‐2α^f/f^;CreER* mice (Table 6 *B*). This is consistent with the reported importance of PHD2/HIF‐2α in erythropoietic control (Arsenault *et al*. 2013; Franke *et al*. 2013), but confirms that erythropoietic stimulation and changes in ventilatory control occur independently.

**Table 6:** **Haematocrit values**

(*A*)	
	Haematocrit (%)
*Phd2^f/f^*	44 ± 4
*Phd2^f/f^;CreER*	79 ± 2****
*Phd2^f/f^*;*Hif‐1α^f/f^;CreER*	78 ± 4****
*Phd2^f/f^*;*Hif‐2α^f/f^;CreER*	53 ± 9§§§
Wild‐type	51 ± 1
*Phd2^+/−^*	53 ± 1
*Phd2^+/−^*;*Hif‐1α^+/−^*	56 ± 1**, §
*Phd2^+/−^*;*Hif‐2α^+/−^*	49 ± 1§
**(*B*)**	
	**Post‐CH haematocrit (%)**
*Hif‐1α^f/f^*	62 ± 2
*Hif‐1α^f/f^;CreER*	61 ± 1
*Hif‐2α^f/f^*	62 ± 0.5
*Hif‐2α^f/f^;CreER*	49 ± 0.5**
Wild‐type	63 ± 4
*Hif‐1α^+/−^*	61 ± 4
Wild‐type	60 ± 1
*Hif‐2α^+/−^*	59 ± 3

*A*, mean haematocrit values (± SEM) in age‐matched male mice; values were measured 6 weeks after starting tamoxifen treatment for conditional mice. *B*, mean haematocrit values (± SEM) after 7 days at 10% oxygen (after chronic hypoxia, CH) (starting 10 days after tamoxifen treatment for the conditional mice). Data were analysed by Tukey's multiple comparison test; significantly different from wild‐type or tamoxifen‐treated *Phd2^f/f^* or *Hif‐2α^f/f^* control mice: **P < 0.01, ****P < 0.0001; significantly different from tamoxifen‐treated *Phd2^f/f^;CreER* or *Phd2^+/−^* mice: ^§^ *P* < 0.05; ^§§§^ *P* < 0.001; *n* = 6–13 mice per genotype.

### Essential role of HIF‐2α in hypoxia‐induced cell proliferation within the CB

Increased ventilatory sensitivity after hypoxic exposure has been associated with growth of the CB in a range of species (Kay & Laidler, 1977; McGregor *et al*. 1984). CBs from *Phd2^+/−^* mice show hyperplasia in the absence of hypoxic exposure, which may contribute to the exaggerated HVR (Bishop *et al*. 2013). We therefore analysed the role of HIF‐α isoforms in the cellular proliferative response to hypoxia in CBs by measuring BrdU incorporation during hypoxic exposure (10% oxygen for 7 days) (as described by Pardal *et al*. 2007; Bishop *et al*. 2013). Responses were compared in mice with either constitutive or conditional loss of HIF‐1α or 2α.

**Figure 5:**
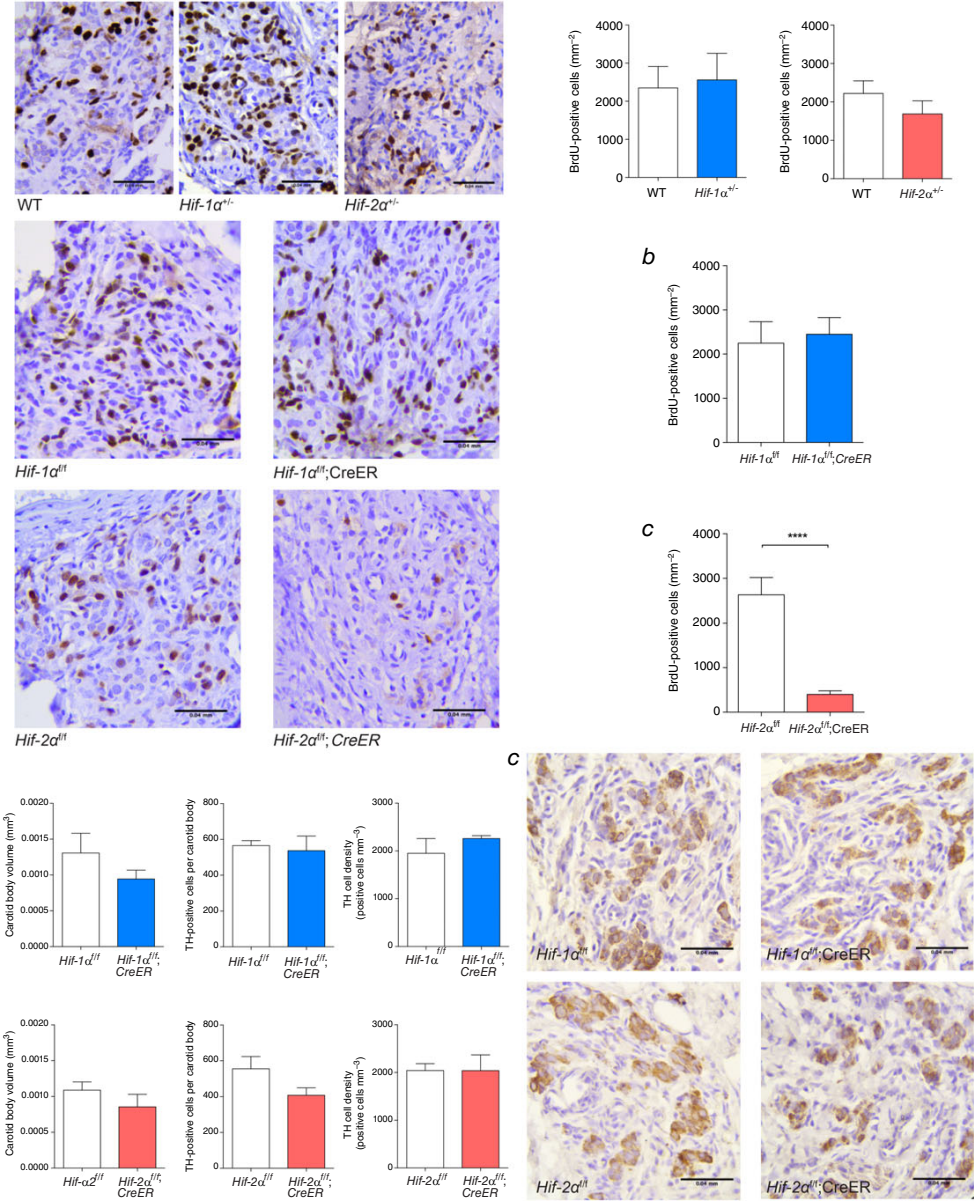
**Hypoxia‐induced cellular proliferation and CB morphology in mice with conditional or constitutive inactivation of *Hif‐1α* or *Hif‐2α*** Immunostaining for BrdU performed on CB sections from: *A*, *Hif‐1α^+/−^* or *Hif‐2α^+/−^* compared to wild‐type (WT) littermates; and *B*, tamoxifen‐treated *Hif‐1α^f/f^;CreER* or *Hif‐2α^f/f^;CreER* compared to *Hif‐1α^f/f^* or *Hif‐2α^f/f^* littermate controls. All mice were treated with BrdU and exposed to 10% oxygen for 7 days prior to being killed and preparation of tissues. Representative images from each genotype are shown in *Aa* and *Ba* where BrdU‐positive cells are stained brown (scale bar = 0.04 mm). *Ab–c* and *Bb–c*, Quantification of BrdU‐positive cells in CBs of *Hif‐1α^+/−^* (*Ab*) or *Hif‐2α^+/−^* (*Ac*) compared to WT littermates and tamoxifen‐treated *Hif‐1α^f/f^;CreER* (*Bb*) or *Hif‐2α^f/f^;CreER* (*Bc*) compared to controls of similar genotype but lacking the CreER allele (*n* = 6 pairs per genotype). *C*, immunostaining for tyrosine hydroxylase (TH) performed on CB sections from *Hif‐1α^f/f^;CreER* or *Hif‐2α^f/f^;CreER* compared to *Hif‐1α^f/f^* or *Hif‐2α^f/f^* littermate mice, 8–9 weeks after tamoxifen treatment. Morphometric analysis of CB volume, TH‐positive cell number and TH‐positive cell density in CBs of *Hif‐1α^f/f^*;CreER (*Ca*) or *Hif‐2α^f/f^;CreER* (*Cb*) compared to controls lacking the CreER allele (*n* = 3 pairs per genotype). *Cc*, representative images from each genotype with TH‐positive cells stained brown (scale bar = 0.04 mm). Values shown are mean ± SEM; ****P < 0.0001.

In control mice (wild‐type, or tamoxifen‐treated *Hif‐1α^f/f^* or *2α^f/f^* mice without the Cre allele), striking incorporation of BrdU was observed after 7 days at 10% oxygen, consistent with previous descriptions (Pardal *et al*. 2007; Bishop *et al*. 2013). Acute conditional inactivation of HIF‐2α resulted in near total loss of BrdU staining, which was reduced to the low background levels observed in normoxic animals (Fig. 5 *B*). In contrast, conditional inactivation of HIF‐1α had no discernible effect (Fig. 5 *B*). Effects of heterozygous loss of HIF‐α were less striking: with HIF‐1α no discernible effect was apparent, whereas with HIF‐2α, reduced BrdU staining was observed that did not reach statistical significance (Fig. 5 *A*). The effect of conditional HIF‐1α and HIF‐2α inactivation on CB morphology was further tested in animals that were maintained under normoxia. HIF‐1α or HIF‐2α inactivation did not significantly alter CB volume, Type I cell number or Type I cell density, and tissues from all genotypes appeared to have qualitatively normal histological structure (Fig. 5 *C*).

## Discussion

Our findings indicate that, despite the long‐term changes in CB morphology that are associated with constitutive heterozygous inactivation of PHD2, acute conditional inactivation of components of the PHD–HIF system in the adult have very major effects on ventilatory responses to hypoxia.

Specifically, inducible inactivation of PHD2 increased ventilatory sensitivity to hypoxia, whereas combined inducible inactivation of PHD2 and HIF‐2α compensated for this effect, and inducible inactivation of HIF‐2α largely ablated enhanced ventilatory sensitivity after chronic hypoxic exposure. These effects on ventilatory sensitivity were large and extended, in the case of inducible HIF‐2α inactivation, to a significant reduction in basal HVR. By contrast, the effects of inducible inactivation of HIF‐1α were modest.

Our work does not exclude developmental or longer term effects of the PHD–HIF system on ventilatory sensitivity to hypoxia, but the effects of acute conditional inactivation were as large as, or larger than, those of constitutive heterozygous inactivation of different components, possibly reflecting the potential for biallelic inactivation in the inducible models. As with the studies of combined inactivation of *Phd*2 and *Hif‐α* genes in the acute model, heterozygous inactivation of HIF‐2α had substantially greater effects on the enhanced HVR in *Phd2^+/−^* animals than heterozygous inactivation of HIF‐1α. In each model effects of HIF‐2α inactivation on the enhanced HVR observed after chronic hypoxia, and after PHD2 inactivation, were similar, strongly supporting the hypothesis that effects of hypoxia are mediated by PHD2 activity on HIF‐2α.

The importance of HIF‐2α in these responses is consistent with high expression of this isoform in sympatho‐adrenal tissues (Tian *et al*. 1998). It is also consistent with findings in human monogenic diseases and analyses of associated phenotypes in mouse models of the relevant human mutations. Thus, the hypomorphic *Vhl* allele associated with Chuvash polycythaemia, and with increased ventilatory sensitivity to hypoxia, manifests somewhat greater dysregulation of HIF‐2α than HIF‐1α (Ang *et al*. 2002; Smith *et al*. 2006; Hickey *et al*. 2010). Similarly, ‘knock‐in’ mouse models of inherited human polycythaemia [‘loss of function’ *Phd2^P249R/+^* (Arsenault *et al*. 2013) and ‘gain of function’ *Hif‐2α^G536W/G536W^* (Tan *et al*. 2013) mice] manifest altered (albeit more minor) effects on ventilatory sensitivity consistent with our findings.

Other work has emphasised the importance of HIF‐1α, at least in the setting of constitutive heterozygous inactivation of *Hif‐α* alleles. This work described reduced VAH in *Hif‐1α^+/−^* animals following 72 h under hypobaric hypoxia (0.4 atmospheres) (Kline *et al*. 2002; Yuan *et al*. 2013). Although trends towards reduced VAH following inactivation of HIF‐1α were observed in several of our datasets, these did not reach significance in any one comparison, and were generally much less than those following inactivation of HIF‐2α. In contrast with our findings, Peng *et al*. (2011) describe increased sensory discharges in hypoxia from CBs isolated from *Hif‐2α^+/–^* mice and ventilatory instability with sighing and apnoea in the intact animals. They also describe an interplay between HIF‐1α heterozygosity and HIF‐2α heterozygosity, such that double heterozygosity for HIF‐1α and HIF‐2α compensated for the effects of HIF‐1α heterozygosity on ventilatory sensitivity (Yuan *et al*. 2013). We are not clear as to the reasons for these differences from our findings. Although we did not observe ventilatory instability in our *Hif‐2α^+/−^* animals, it is possible that such a phenotype might be related to differences in background, and might obscure, or even reflect, the abnormalities of ventilatory control in response to hypoxia that we observed.

One difference in our studies was the use of 10% oxygen with 3% carbon dioxide to compensate for hyperventilation‐induced hypocapnia. This stimulus was used instead of the computer‐regulated end‐tidal clamping of carbon dioxide that has been used to clarify the measurement of ventilatory sensitivity to hypoxia in humans (Howard & Robbins, 1995), but is not feasible in mice. Our calculations indicated that, at the levels of hyperventilation that were induced, this manoeuvre provides approximate compensation for hypocapnia, and thus much more closely induces pure hypoxaemia than simple inhalation of 10% oxygen. Consistent with results in humans, HVRs were much better sustained when these attempts to compensate for hypocapnia were made than when they were not, and the effects of interventions on the size of the HVR were more clearly seen. Nevertheless, we cannot exclude that some animals have been subjected to an additional hypercapnic stimulus under these conditions, potentially acting outside the CB. We therefore analysed HVRs both in response to 10% oxygen with 3% carbon dioxide and in response to 10% oxygen alone, and have provided all datasets. In general, responses to both stimuli showed similar trends from interventions, although responses to 10% oxygen were smaller and more variable and the effects of interventions less frequently reached statistical significance.

Importantly, however, because ventilatory sensitivity in mice is reported to vary with genetic background (Han *et al*. 2002; Balbir *et al*. 2007), our observations on the dominant importance of PHD2 and HIF‐2α were observed clearly using differently derived inactivating HIF‐α isoforms and on the cellular proliferation that is observed in the CB in response to sustained hypoxia. Hypoxia‐induced cellular proliferation as assessed by BrdU staining was specifically, and almost totally, ablated by acute inactivation of *Hif‐2α*. In contrast, CBs from animals with either acute or constitutive heterozygous inactivation of *Hif‐1α* were indistinguishable from controls.

Although changes in HVR and in these proliferative responses were altered in parallel, it is not yet precisely clear how they are connected. Nevertheless, quantitative differences in the effects of HIF‐2α heterozygosity on HVR and on cellular proliferation, taken together with the rapid (within hours) effects of sustained hypoxia on HVR (reviewed by Robbins, 2007), suggest that enhanced HVR is not simply a reflection of increased CB mass. Further work will be required to understand how the role(s) of HIF‐2α in each process are interlinked. Interestingly, cellular proliferation within the CB has been demonstrated to depend on an interplay between the Type I neurosecretory ‘glomus’ cells and Type II ‘supporting’ cells (Pardal *et al*. 2007; Platero‐Luengo *et al*. 2014). Proliferation originates in stem‐like Type II cells that then develop into Type I cells (Pardal *et al*. 2007), but is dependent on paracrine responses from hypoxia‐activated Type I cells (Platero‐Luengo *et al*. 2014). Although the rapid electrophysiological events that activate Type I cells in response to hypoxia are believed to be independent of HIF and HIF hydroxylase activity, our data clearly indicate that the integrity of HIF‐2α is required at some stage in the events linking hypoxic exposure to proliferation and differentiation of cells within the CB.

It is also likely that other components of the HIF hydroxylase system are important in defining CB morphology through developmental or adaptive processes. For instance, inactivation of PHD3 leads to altered CB cell morphology, probably through defective developmental culling of sympathoadrenal neurones (Bishop *et al*. 2008), but *Phd3^−/−^* mice show normal HVR (Bishop *et al*. 2013). Furthermore, inactivation of VHL in Type I cells by TH‐driven Cre recombinase results in atrophy of the CB (Macias *et al*. 2014), suggesting either that the very high levels of HIF‐α associated with complete inactivation of VHL impair CB cell viability or that some other

consequence of defective VHL function has the same effect.

Nevertheless, despite these complexities, the current work reveals the importance of specific components of the HIF hydroxylase system (the PHD2 – HIF‐2α enzyme–substrate couple) in mediating both anatomical and physiological responses that underlie ventilatory acclimatisation to hypoxia.

## Additional information

### Competing interests

C.W.P. and P.J.R. are scientific co‐founders and hold equity in ReOx Ltd, a university spin‐out company that seeks to develop therapeutic inhibitors of the HIF hydroxylases.

### Author contributions

E.J.H., P.J.T., P.A.R., C.W.P., K.J.B., P.J.R. and T.B. conceived and designed the experiments. E.J.H., L.G.N., P.J.T., R.L., J.W.F., G.D., I.R., P.A.R., C.W.P., K.J.B., P.J.R. and T.B. collected, analysed and interpreted the data. E.J.H., P.J.R. and T.B. drafted the article. E.J.H., L.G.N., P.J.T., R.L., J.W.F., G.D., I.R., P.A.R., C.W.P., K.J.B., P.J.R. and T.B. revised the manuscript critically for important intellectual content. All authors have read and approved the final copy. All experiments were carried out at the University of Oxford.

### Funding

The work was supported by the Wellcome Trust (grant number 091857/Z/10/Z), the Ludwig Institute for Cancer Research and the Medical Research Council (grant number G101134).
